# Supplementary material for: Development of a validated PROM set for children with tinnitus using the Patient-Reported Outcomes Measurement Information System – Protocol for the ‘Tinnitus in Children’ (TinC) study
Source: PLoS One. 2025 Oct 16;20(10):pone.0334549.exml. doi: 10.1371/journal.pone.0334549 (PMC12530566; doi:10.1371/journal.pone.0334549)
Supplement: Attachment — Submitted filename: Response to reviewers.docx [file pone.0334549.s001.docx]

28-09-2025
Dear dr. Mankekar and Reviewer #1,

We thank you for your careful consideration of our manuscript and for providing constructive feedback. We have revised our manuscript to address all points raised.

Regarding the comments from the Academic Editor, we have now revised our manuscript to meet PLOS ONE’s style requirements, including file naming (comment 1). Following the editor’s guidance (comment 2), we have updated our funding statement to clarify that no additional external funding was received. The revised statement reads as follows:
*“This project is partially funded by the Dorhout Mees Stichting and through crowdfunding by Stichting Oorfonds (UMCU budgeted grant funding number 76231.92). However, the funders did not play any role in the study design and preparation of the manuscript. There was no additional external funding received for this study.”*
We have removed our funding statement from the manuscript and only included the updated statement in this letter, so that it can be changed in the online submission (comment 3).
Comment 4 is not applicable, as the reviewer did not suggest citing additional published work. We have verified that our reference list is complete and accurate (comment 5).

Reviewer #1 commented the following:
*“The study needs to emphasize otological conditions such as audiological tests, examination of the ears to rule out otological disease. Radiological imaging in the form of MRI / CT Scan of the temporal bone needs to be included. Exposure to noise and previous otological surgeries need to be considered. Inclusion and exclusion criteria need to be established. If these are not in place then the proposed study will have no impact or relevance.”*

We would like to thank the reviewer for this comment. We agree that audiological tests, otological examinations, and imaging are important when evaluating tinnitus in clinical care or studies with an etiological focus. However, the aim of our study is different. Our focus is on developing a patient-reported outcome measure (PROM) set that captures the impact of tinnitus on children’s daily lives, regardless of underlying causes or clinical characteristics.
For this reason, we apply broad inclusion criteria to ensure that the PROM set will be generalizable and relevant to the full pediatric tinnitus population. Additional clinical measures, as suggested, are therefore not required within the scope of our study. Instead, our inclusion and exclusion criteria ensure that participants are children with tinnitus who can meaningfully share their experiences (e.g., sufficient Dutch language proficiency and no diagnosed cognitive or developmental delays incompatible with age-appropriate functioning).
Once the PROM set is developed and validated, future studies could utilize this tool to explore the impact of tinnitus in children in relation to clinical characteristics. Such studies could provide valuable insights into pediatric tinnitus, but are beyond the scope of the present study.

We appreciate the opportunity to revise our manuscript and hope that the changes and clarifications address the concerns raised. Please let us know if any further modifications are required.

Sincerely,

On behalf of all authors,

Denise Fuchten
